# Increasing skepticism toward potential liars: effects of existential threat on veracity judgments and the moderating role of honesty norm activation

**DOI:** 10.3389/fpsyg.2015.01312

**Published:** 2015-09-01

**Authors:** Simon Schindler, Marc-André Reinhard

**Affiliations:** Department of Psychology, University of KasselKassel, Germany

**Keywords:** veracity judgments, judgmental bias, terror management theory, mortality salience, norm of honesty

## Abstract

With the present research, we investigated effects of existential threat on veracity judgments. According to several meta-analyses, people judge potentially deceptive messages of other people as true rather than as false (so-called truth bias). This judgmental bias has been shown to depend on how people weigh the error of judging a true message as a lie (error 1) and the error of judging a lie as a true message (error 2). The weight of these errors has been further shown to be affected by situational variables. Given that research on terror management theory has found evidence that mortality salience (MS) increases the sensitivity toward the compliance of cultural norms, especially when they are of focal attention, we assumed that when the honesty norm is activated, MS affects judgmental error weighing and, consequently, judgmental biases. Specifically, activating the norm of honesty should decrease the weight of error 1 (the error of judging a true message as a lie) and increase the weight of error 2 (the error of judging a lie as a true message) when mortality is salient. In a first study, we found initial evidence for this assumption. Furthermore, the change in error weighing should reduce the truth bias, automatically resulting in better detection accuracy of actual lies and worse accuracy of actual true statements. In two further studies, we manipulated MS and honesty norm activation before participants judged several videos containing actual truths or lies. Results revealed evidence for our prediction. Moreover, in Study 3, the truth bias was increased after MS when group solidarity was previously emphasized.

## Introduction

As a consequence of the 9/11 terror attacks, President George W. Bush proclaimed the “War on Terror.” In addition to al-Qaeda, this military campaign was also targeted toward Saddam Hussein and Iraq because they were assumed by the US government to possess weapons of mass destruction. In their speeches, Secretary of State Colin Powell and President Bush assured the existence of such weapons, leading most Americans to support the war against Iraq (Gallup and Newport, 2004). However, investigations of a task force did not substantiate this argument (Duelfer, 2004): no weapons of mass destruction were found. Consequently, one third of Americans (Gallup and Newport, 2004) and some authors imputed deliberate deception to President Bush (e.g., Corn, 2004). Whether their accusation holds true or not, this incident clearly illustrates the importance of investigating the effects of existential threats (e.g., 9/11 attacks) on veracity judgments. By referring to findings on terror management theory (TMT; Greenberg et al., 1986), we assume that existential threat affects veracity judgments on potentially deceptive messages depending on which social norm (e.g., honesty) is momentarily salient.

According to TMT (Greenberg et al., 1986), cultural worldviews function as an anxiety buffer against the ever-present terror of potential death by providing a meaningful, orderly conception of reality that contains a set of standards and values. By living up to those cultural standards, people are enabled to believe that they are valuable beings in a meaningful reality. Based on this idea, the mortality salience (MS) hypothesis states that reminding people of their mortality should lead to an increased need for the protection provided by such worldview-based beliefs: people want others to correspond to their own cultural worldview, resulting in derogating those who violate important cultural standards and supporting those who uphold them (for an overview, see, e.g., Greenberg et al., 2008). In the first study of Rosenblatt et al. (1989), for example, judges had to recommend a bond for a prostitute. Results indicated that judges in the MS condition, on average, assigned a much higher bond than judges in the control condition. Moreover, McGregor et al. (1998) indicated that MS enhanced physical aggression toward those who attack one’s political orientation whereby the aggressor had administered to them an increased amount of hot sauce. Thus, this line of research strongly supports the idea that moral principles are part of the cultural anxiety buffer and that transgressions of moral standards enhance devaluation of the transgressors and even desires of punishing them.

However, because cultural standards can be contradictory and result in a mixed pattern of behaviors (e.g., aggression vs. helpfulness), Jonas et al. (2008) connected TMT with the focus theory of normative conduct (Cialdini et al., 1991), stating that norms have to be salient in attention or high in accessibility to influence behavior (see also Higgins and Bargh, 1987). This may be because people habitually follow a norm and/or because certain conditions of the situation itself account for the norm’s salience. In line with this reasoning, MS was found to increase adherence to a broad range of activated norms, such as pacifism and conservatism (Jonas et al., 2008), egalitarianism and helpfulness (Gailliot et al., 2008; Schindler et al., 2014), proenvironmental norms (Fritsche et al., 2010), norms of justice (Jonas et al., 2013; Schindler and Reinhard, 2015), and the norm of reciprocity (Schindler et al., 2012, 2013; Schindler and Reinhard, in press). In sum, TMT research clearly supports the idea that MS increases sensitivity toward the compliance of cultural norms, especially when they are of focal attention. Notably, the influence of activated norms in these studies referred to one’s own norm compliance, that is, MS was assumed to increase the motivation to fulfill the norm by oneself. By contrast, the current research focused on the perception and judgment of other individuals who potentially engaged in behavior conflicting with one’s worldview (i.e., deception).

Despite the large amount of TMT research on worldview defense and norm adherence, to our knowledge, no empirical investigations have been done on the norm of honesty. This seems the more astonishing if one takes into account that surveys have indicated honesty to be one of the most important norms in people’s lives, in general (e.g., Geißler et al., 2013), and specifically, for example, in romantic relationships (Weber and Ruch, 2012) and politics (Bishin et al., 2006). Although literature mentions honesty as an evolutionarily developed form of social capital that can be accumulated (e.g., Somanathan and Rubin, 2004), for the current work, we refer to honesty as a norm; namely, that one should tell the truth and should not lie.

Although lying has always been a social issue (Ekman, 1992), people’s ability to discriminate accurately between lies and truths is not particularly well-developed. In a comprehensive meta-analysis of more than 200 studies, Bond and DePaulo (2006) found that people without special training were slightly better than the chance result of a coin toss (54%) when judging the veracity of actual true or deceptive statements (for similar results, see Ekman and O’Sullivan, 1991; Aamodt and Custer, 2006; Hartwig and Bond, 2011). Besides discrimination accuracy, Bond and DePaulo additionally found that people were better at correctly identifying truths as non-deceptive (61%) than they were at identifying lies as deceptive (47%; so-called *veracity effect*). Based on parallel findings of his meta-analysis, Vrij (2000) asserted that analyzing accuracy at detecting actual lies separately from detecting actual truths revealed “that people are particularly poor at detecting lies” (Vrij, 2000, p. 240). This result is due to people’s general tendency to judge messages as true (so-called *truth bias*): Bond and DePaulo’s analysis of percentage truth classifications revealed a mean of about 57%, which differed significantly from 50%, supporting the truth bias. Thus, when people are truth biased—that is, when they more frequently judge messages as true rather than as false—logically (or mathematically), they are likely to be better at correctly judging actual truths compared to lies (e.g., Zuckerman et al., 1979; Levine et al., 2010). For example, given that 10 statements out of twenty are actually false, the accuracy for actual false statements is 100% if all statements are judged as false. Given this mathematical relationship, judgmental bias actually determines the probability of detecting liars. Notably, overall-discrimination accuracy was found to be unrelated to the truth bias (Bond and DePaulo, 2006; Hartwig and Bond, 2011).

Literature attributes the truth bias to the phenomenon whereby in daily communications, people usually believe messages from other people without questioning honesty (e.g., Levine et al., 2010). Research has shown the truth bias to be affected by various factors: it is increased, for example, in face-to-face interactions (Buller et al., 1991) or in close relationships (McCornack and Parks, 1986). Moreover, truth bias in close relationships has been shown to decrease when there are contextual cues for suspicion (McCornack and Levine, 1990), that is, when beliefs about communicative honesty are questioned. Judgmental biases have also been shown to vary between different groups: the truth bias can, for example, be found for teachers (e.g., Reinhard et al., 2011a). By contrast, prisoners (e.g., Bond et al., 2004, 2005) and police officers (e.g., Meissner and Kassin, 2002; Masip et al., 2005) showed a *lie bias*, that is, the tendency to judge messages as false.

By applying signal detection theory (Green and Swets, 1966), these differences in judgmental bias have been discussed and found to depend on how people weigh the error of judging a true message as a lie (false alarm) and the error of judging a lie as a true message (miss). For example, it seems much worse for a police officer to believe a liar than to suspect somebody who tells the truth. By contrast, in daily conversations, falsely blaming a friend of telling a lie might lead to an end of the friendship. Thus, judgmental biases can plausibly be explained by unequal importance to the two types of error (see also Hurst and Oswald, 2012). Based on the idea that possible costs of making a judgmental error vary as a function of situational variables, Hurst and Oswald (2012) experimentally manipulated error weighting. Results provided initial evidence for the idea that an increased salience of the consequences of a false alarm (vs. a miss) results in a truth bias.

Traditionally, research on veracity judgments has rather neglected the underlying motivational processes of judgmental biases (Levine et al., 1999, 2010; Levine and McCornack, 2001; Masip et al., 2009). However, taking into account that people’s judgmental biases actually determine the probability of detecting actual liars, motivational aspects in error weighing seem to play a crucial role.

In the current research, we address the effect of existential threat on judgmental bias depending on norm activation. In general, people tend to judge messages of others as true. Even in laboratory settings—where suspicion is increased by mentioning the potential occurrence of deceptive statements—a truth bias is likely to be found (e.g., Bond and DePaulo, 2006). According to signal detection theory, the truth bias is based on the normally high aversion toward the error of judging an actual message as a lie (false alarm = error 1). Reducing the weight of this type of error should therefore lead to a reduced truth bias (Hurst and Oswald, 2012). Additionally, increasing the motivation to avoid the error of judging an actual lie as true (miss = error 2) should also lead to a reduced truth bias, even possibly resulting in a lie bias (e.g., in the case of police officers). We now propose that error weighing is affected by MS and honesty norm activation. MS was previously shown to increase people’s motivation to devaluate and to even punish moral transgressors (e.g., McGregor et al., 1998). Moreover, research evidenced MS to increase the motivation to fulfill cultural norms, especially when they had been activated (e.g., Jonas et al., 2008). Regarding one’s own norm compliance, it is plausible to assume that MS increases honest behavior, especially when the norm of honesty has been activated. However, instead of investigating one’s own norm compliance, the current research focuses on the perception of potential worldview transgressors, specifically the perception of persons who potentially engaged in deceptive behavior. It is important to recognize that this situation already implies some violation of the norm of honesty. Therefore, an increased level of suspicion can be assumed (McCornack and Levine, 1990). Activating the honesty norm in a situation where dishonesty is present should increase a defensive reaction, especially after MS because MS increases sensitivity toward norm violations (Rosenblatt et al., 1989). Consequently, the aversion toward error 1 (false alarm) should be reduced. We tested this assumption in a pilot study (Study 1). Based on this assumed shift in error weighing, we hypothesized that when activating the honesty norm, people show a reduced truth bias when being reminded of their own death (vs. control group). Given that the number of judged messages as true (or false) is mathematically linked to accuracy of actual true or false statements (e.g., Levine et al., 2010), respectively, we expected a reduced truth bias to result in better detection accuracy of actual lies and in worse detection accuracy of actual true messages. In Studies 2 and 3, we manipulated MS and honesty norm activation (by explicitly emphasizing its social importance) before participants watched and judged several different sets of videos containing actual true or false messages. In Study 2 (online study), we used a no-activation control condition, whereas in Study 3, a group solidarity activation condition was included. In this case, people under MS should strive to enhance solidarity by avoiding error 1, that is, by avoiding convicting people of lying. Therefore, we predicted an increased truth-bias under MS (vs. control group), resulting in worse (better) detection accuracy of actual deceptive (true) messages. We explicitly want to emphasize that the predicted accuracy effects are simply mathematically linked to shifts in the truth bias and have nothing to do with any ability effects because they would refer to an overall improvement in discriminating true statements from lies. Additionally, there is substantial evidence showing that norm activation affected attitudes and behavior only when additional motivation (such as MS) was induced (e.g., Jonas et al., 2008; Fritsche et al., 2010; Schindler et al., 2013). We therefore did not expect an effect of norm activation on veracity judgments in our studies when mortality is not salient.

## Study 1

Before investigating the hypothesized effects regarding the truth bias, we wanted to check the underlying assumption that MS, in combination with activating the norm of honesty, actually decreases the weight of error 1; that is, that under those circumstances, falsely blaming an innocent person of being deceitful is perceived as less serious. Therefore, in this pilot study, for those participants who read a short statement about the social importance of the honesty norm, we expected a lower weight of error 1 after MS (vs. control group).

### Material and Methods

#### Subjects and Design

Ninety nine US citizens (53 women; *M*_age_ = 35.84, *SD* = 12.23) participated in our Internet study via Amazon Mechanical Turk (cf. Buhrmester et al., 2011). All participants were randomly assigned to the experimental conditions in a 2 (MS vs. TV control condition) × 2 (honesty vs. no-activation control condition). Prior to participating, all subjects in this study (also in Studies 2 and 3) had given written informed consent in line with APA’s Ethical Principles of Psychologists and Code of Conduct.

#### Procedure and Measures

After the demographic measures, participants received the MS or TV control treatment (see, e.g., Jonas et al., 2008): they were asked to write down the first sentence that came to their mind when they thought about their own death (MS condition) or about watching TV (control condition). When using such explicit death primes, a distractor is necessary to elicit effects of worldview defense and bolstering (Arndt et al., 2002). Therefore, as in many studies on TMT (Burke et al., 2010), participants filled out the Expanded Form of the Positive and Negative Affect Schedule (PANAS-X; Watson and Clark, 1992)^1^. Next, participants in the honesty norm activation condition read a short statement about the fundamental importance for society’s stability to follow the norm of honesty. In the no-activation control condition, this statement did not occur. Then, to maintain the cover story, participants were instructed to watch several messages of students describing movies they really liked or disliked, and that some of these messages were in fact true because the reporting students either liked or disliked the movie (for detailed information, see Study 2). On the next page, participants then read that before they would watch the videos, we would be interested in their opinion on false accusations in general. To measure the weight of error 1, they were asked to rate the following statement on a seven-point scale (1 = “not serious at all,” 7 = “very serious”): “In everyday life, how serious do you regard mistaking a truthful statement for a deceitful one generally?” After having answered this question, participants were told that this had been a pre-test and that they would not watch any videos. Finally, they were thanked and debriefed.

### Results and Discussion

The mean weight of error 1 was 4.79 (*SD* = 1.39). We conducted a 2 (Salience: MS vs. TV control condition) × 2 (Norm activation: honesty vs. no-activation control condition) analysis of variance (ANOVA), with weight of error 1 as the dependent variable. No main effects occurred. However, as predicted, the interaction between salience and norm activation was significant, *F*(1,95) = 3.95, *p* = 0.050, $\eta_{p}^{2}$ = 0.04. The follow-up simple effects analyses within norm activation revealed that, in line with our hypothesis, when honesty was activated, error 1 was perceived as significantly less serious in the MS condition (*M* = 4.23, *SD* = 0.97) compared to the TV control condition (*M* = 5.15, *SD* = 1.32), *F*(1,95) = 5.33, *p* = 0.023, *d* = 0.79. In the no-activation control condition, no effect of salience occurred, *F* < 1, *p* = 0.642. Moreover, simple effects analyses within the salience condition revealed that participants perceived error 1 as marginally significantly less serious after MS than did participants in the no-activation control condition (*M* = 4.93, *SD* = 1.34), *F*(1,95) = 3.14, *p* = 0.080, *d* = 0.58. In the TV control condition, there was no significant effect of norm-activation, *p* = 0.307.

In sum, this pilot study provides initial evidence for our idea that MS affects error weighing, depending on norm activation. Specifically, we predicted and found that compared to all other conditions, participants perceived error 1 as least serious when the norm of honesty was pronounced and mortality was salient. We interpret this result as a sign of reduced aversion toward false alarms when judging potentially deceptive messages. This should further be linked to a reduction in truth bias.

## Study 2

In this online study, participants had to judge the veracity of several videos in which other people talk about movies or series they ostensibly like or dislike. For participants who read a short statement about the socially important norm of honesty, we assumed MS (vs. control group) to decrease aversion of false alarms. This should result in judging the messages less frequently as true, which should logically (i.e., mathematically) lead to better detection accuracy when judging actual lies and to worse detection accuracy when judging actual true statements. In the no-activation control condition, we did not expect an effect of MS. If anything, based on the general finding that MS increases the motivation to bolster one’s worldview (e.g., Greenberg et al., 2008), one could speculate that MS increases the importance of avoiding a false alarm, resulting in an increased truth bias.

### Material and Methods

#### Subjects and Design

We calculated the sample size to obtain sufficient power to detect both the two-way interaction on the judgmental bias and the three-way interaction on detection accuracy (80% to detect an effect if one exists; Cohen, 1988). Power analysis assuming an effect of the predicted three-way interaction on detection accuracy of *f* = 0.20 (cf. Reinhard and Schwarz, 2012), and a small correlation between the dependent variables, revealed an *N* of 120. Given the possibility of easily obtaining large samples through Internet experiments (Reips, 2002), participants in this Internet study included 156 German people (116 women; *M*_age_ = 21.67, *SD* = 2.24). The design was a 2 × 2 × 2 mixed model design with salience (MS vs. TV control condition) and norm activation (honesty vs. no-activation control condition) as between-participants variables, and type of message (lie vs. truth) as the within-participants variable.

#### Procedure and Measures

Parallel to the pilot study, after the demographic measures, participants received the MS or TV control treatment. Then, participants filled out the PANAS (Watson et al., 1988). Next, participants in the honesty norm activation condition read a short statement about the fundamental importance for society’s stability to follow the norm of honesty. In the no norm activation control condition, this statement did not occur. Then, participants were instructed to watch 24 messages of students describing movies they really liked or disliked, and that some of these messages were in fact true, as the reporting students did like or dislike the movie (for detailed description of the stimulus material fabrication, see Reinhard and Schwarz, 2012, Study 2). They were also told that some of these messages were not true because the students described a movie they liked (disliked) as though they disliked (liked) it. Participants were further told to put themselves in the position of the person who interviewed the students about their attitudes. Each participant was presented with one of the three sets of 24 messages. Then they saw each of the 24 messages, and immediately after watching each message, participants had to classify it as a lie or as true. After having judged all messages, participants were thanked and debriefed.

### Results

#### Truth Bias

**FIGURE 1:**
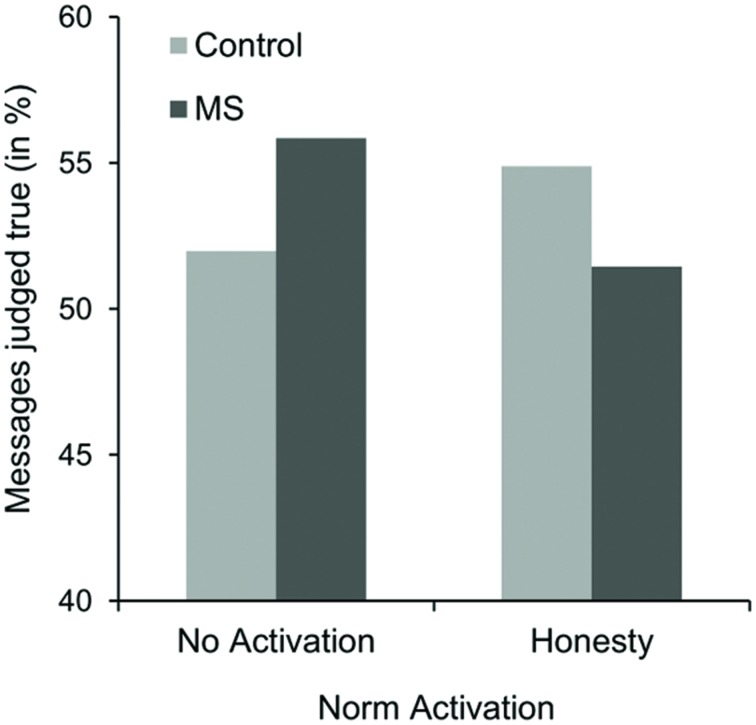
**Means of messages judged true (in %) as a function of norm activation and salience condition in Study 2**.

We conducted a 2 (Salience: MS vs. TV control condition) × 2 (Norm activation: honesty vs. no-activation control condition) ANOVA, with the number of messages judged true (in %) as the dependent variable^2^. Overall, participants classified 53.39% (*SD* = 10.19) of the messages as true. This value is significantly different from 50%, *t*(155) = 4.16, *p* < 0.001. Results of the ANOVA revealed no main effects, both *F*s < 1. However, as predicted, a significant interaction effect of salience and norm activation condition occurred, *F*(1,152) = 5.01, *p* = 0.027, $\eta_{p}^{2}$ = 0.03^3^. As expected, when the importance of honesty was pronounced, percentages of messages judged true were not significantly different from 50% in the MS condition (*M* = 51.46%, *SD* = 9.69), *t*(39) = 0.95, *p* = 0.347, whereas a truth bias occurred in the TV control condition (*M* = 54.90%, *SD* = 9.62), *t*(39) = 3.22, *p* = 0.003 (see **Figure 1**). Although these results are in line with our hypotheses, simple effects analyses revealed that percentages of messages judged true in the honesty condition were not significantly lower in the MS condition compared to the TV control condition, *F*(1,152) = 2.31, *p* = 0.131. However, we would like to note that the *p*-value is based on a two-sided test. Thus, given our directional hypothesis, the difference appears to be at least marginally significant. Additionally, and in line with our argument that activating the norm of honesty should be responsible for this effect, the truth bias still occurred under MS in the no norm activation control condition (*M* = 55.86%, *SD* = 9.34), *t*(31) = 3.55, *p* = 0.001. Simple effects analyses revealed that percentages of messages judged true in the MS condition were marginally significantly lower in the honesty condition compared to the no norm activation control condition again, *F*(1,152) = 3.36, *p* = 0.069. When the norm of honesty was not activated, percentages of messages judged true failed to be significantly different from 50% in the TV control condition (*M* = 51.99 %, *SD* = 11.42), *t*(43) = 1.156, *p* = 0.254. For this condition, simple effects analyses revealed that percentages of messages judged true were however not significantly lower in the MS condition compared to the TV control condition, *F*(1,152) = 2.71, *p* = 0.102. Finally, simple effect analysis within the TV control condition revealed no significant effect of norm activation, *F*(1,152) = 1.73, *p* = 0.190.

#### Detection Accuracy

We conducted a 2 (Salience: MS vs. TV control condition) × 2 (Norm activation: honesty vs. no-activation control condition) × 2 (Type of message: lie vs. true) mixed-model design ANOVA, with detection accuracy (in %) as the dependent variable. Salience and norm activation were between-participants variables, and type of message was a within-participants variable.

**FIGURE 2:**
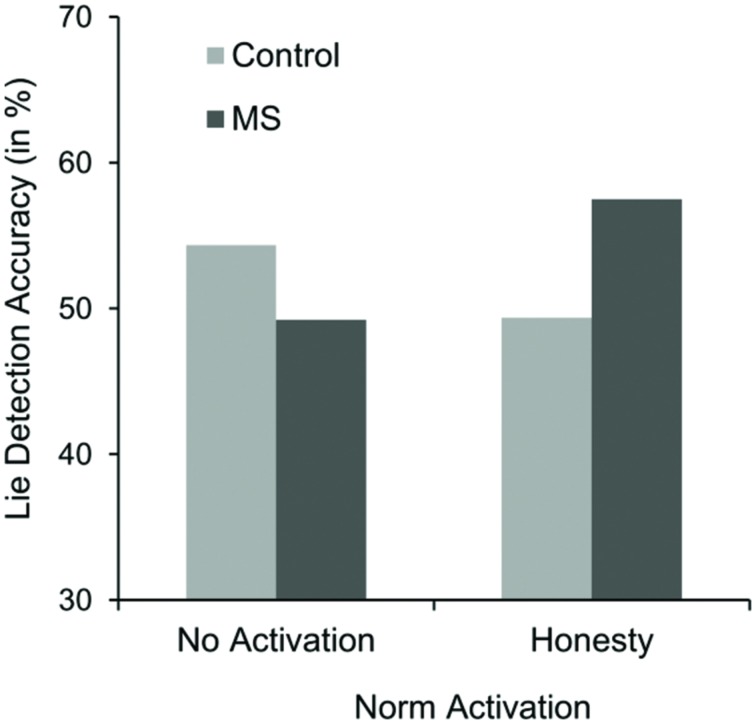
**Means of detection accuracy of actual lies (in %) as a function of norm activation and salience condition in Study 2**.

Overall, the mean percentage of correct lie-truth classifications was 56.22% (*SD* = 11.53). On average, participants were significantly better than chance in their lie-truth classifications, *t*(155) = 6.74, *p* < 0.001. Results of the mixed-model design ANOVA revealed a main effect of the type of message, indicating that participants were better at classifying truthful messages as actually true (*M* = 59.62%, *SD* = 13.82) than they were at classifying deceptive messages as actual lies (*M* = 52.83%, *SD* = 16.81), *F*(1,152) = 18.92, *p* < 0.001, $\eta_{p}^{2}$ = 0.11 (veracity effect). Furthermore, the predicted three-way interaction between salience and norm activation and type of message occurred, *F*(1,152) = 5.01, *p* = 0.027, $\eta_{p}^{2}$ = 0.03. Simple effects analyses for detection accuracy for actual lies within the honesty condition revealed that participants under MS were better (*M* = 57.50%, *SD* = 13.84) compared to the TV control condition (*M* = 49.38%, *SD* = 19.92), *F*(1,152) = 6.44, *p* = 0.012 (see **Figure 2**). Additionally, and in line with our argument that activating the norm of honesty should be responsible for this effect, simple effects within the MS condition showed that participants in the honesty condition were also better at detecting actual lies (*M* = 57.50%) than participants in the no-activation control condition (*M* = 49.22%), *F*(1,152) = 5.95, *p* = 0.016. Simple effects within the no-activation control condition indicated that participants under MS (*M* = 49.22%, *SD* = 14.72) were not significantly worse compared to participants in the TV control condition (*M* = 54.36%, *SD* = 16.91), *F*(1,152) = 2.39, *p* = 0.124. Regarding detection accuracy for actual true messages, no effects occurred (accuracy ranged from 58.33 to 60.94%), all *F*s < 1.

#### Mediation of Lie Detection Accuracy

The regression analyses supported the hypothesized mediation (i.e., the mathematical link) of percentage of messages judged true on detection accuracy of actual lies. The interaction of Salience (TV control condition = -1, MS condition = 1) × Norm Activation (no-activation condition = -1, honesty condition = 1) predicted percentage of messages judged true in step one, *b* = -1.83, SE *b* = 0.82, *t*(152) = -2.24, *p* = 0.027. In step two, the percentage of messages judged true predicted participants’ detection accuracy, *b* = -12.24, SE *b* = 0.93, *t*(151) = -13.19, *p* < 0.001. In step three, the total effect of the Salience × Norm activation interaction on detection accuracy of actual lies, *b* = 3.32, SE *b* = 1.34, *t*(152) = 2.48, *p* = 0.014, was reduced to non-significance when accuracy was regressed on the interaction and percentage of messages judged true, *b* = 1.12, SE *b* = 0.93, *t*(151) = 1.21, *p* = 0.230. Additionally, bootstrapping the proposed mediated moderation (using Model 8 of the Process macro of Hayes, 2013, and 5000 samples) also revealed significant results, that is, the 95% confidence interval did not include zero [0.74, 8.26].

### Discussion

Results of this study support our interaction hypothesis that when pronouncing the norm of honesty, existential threat attenuates the truth bias when judging potentially deceptive messages of others. Mediational analyses further indicated that this effect is mathematically linked to an increase of the accuracy rate when judging actual lies. Thus, when people less (more) frequently judged messages as true rather than as false, they automatically improved (worsened) at correctly judging actual deceptive statements. Somewhat unexpectedly, we found no effects on actually true messages (see General Discussion).

In this study, no truth bias occurred in the baseline condition (i.e., no-activation in the TV control condition). This is in contrast to what we would have expected. Consequently, comparing the judgmental bias within the TV control condition revealed that norm activation affects truth bias by trend. However, given that this effect is not significant, it should not be overstated, especially, because it is theoretically hard to explain. Furthermore, regarding the condition where no norm was activated, MS, by trend, increased the truth bias. Besides the given possibility of measurement error in the baseline condition, one could also think of a theoretical explanation. According to TMT, group membership and social identity plays a crucial role when confronted with one’s own death (e.g., Greenberg et al., 1990). Taking into account that people in the videos were introduced as students, and that most of the participants in the current study (about 78%) had reported being students too, it could be that the increased truth bias after MS can be interpreted as some kind of ingroup bias. That is, MS might increase people’s motivation to believe that ingroup members do not lie to each other. We will test this assumption in more detail in Study 3.

## Study 3

To bolster the findings of Study 2, we designed a laboratory study applying video material which contained true or false messages of students who had been interviewed about having cheated on a test. Parallel to Study 2, we assumed that participants who read a statement about the importance of honesty should show a decreased truth bias under MS (vs. control group), which should consequently lead to better (worse) detection accuracy when judging actual deceptive (true) messages. Additionally, to investigate the effect in the no-activation control condition in Study 2, we included a solidarity activation condition. As mentioned above, group membership and social identity are highly relevant after MS: empirical evidence demonstrates existential threat to enhance striving for togetherness and group attachment (e.g., Mikulincer et al., 2003), in-group favoritism (e.g., Harmon-Jones et al., 1996; Jonas et al., 2002), and out-group derogation (e.g., Greenberg et al., 1990; Fritsche et al., 2008). We, therefore, assumed pronouncing the importance of group solidarity under MS to increase motivation to avoid false alarms, leading to increased truth bias, which should further result in worse (better) detection accuracy of actual deceptive (true) messages.

### Material and Methods

#### Subjects and Design

We calculated the sample size to obtain sufficient power to detect both the two-way interaction on the judgmental bias and the three-way interaction on detection accuracy. Power analysis, assuming a medium effect of the predicted three-way interaction of *f* = 0.25 (cf. Marksteiner, 2013) and a small correlation between the dependent variables, revealed an *N* of 78. Participants in this study included 81 students (46 women) recruited on the campus of a German university (*M*_age_ = 22.90, *SD* = 2.45). The design was a 2 × 2 × 2 mixed model design, with salience (MS vs. dental pain control condition) and norm activation (honesty vs. solidarity condition) as between-participants variables, and type of message (lie vs. truth) as a within-participants variable.

#### Procedure and Measures

After the demographic measures, participants in the MS condition answered two open-ended questions about death, whereas participants in the control condition answered two open-ended questions about dental pain (e.g., Rosenblatt et al., 1989). Then, they filled out the PANAS. Next, participants in the honesty condition read an alleged excerpt from the university’s code of ethics in which the norm of honesty and the responsibility of every student to uncover academic misbehavior were pronounced. By contrast, in the solidarity activation condition, the importance of cohesion and the responsibility of every student to contribute to a companionate togetherness without jealousy were pronounced. Afterward, participants were instructed to watch eight messages of students being interviewed about being accused of having cheated on a test, due to their excellent performance. To produce this stimulus material, in the cheating condition, students had been persuaded by a confederate to use forbidden additives (e.g., calculator) after the attendant had left the room to answer an alleged phone call. Participants were told that all students pretended to have not been cheating and that some of these messages were, in fact, true. Additionally, they were informed that some of these messages were not true because some of the students pretended to have not been cheating even though they did cheat (for a similar procedure, see Exline et al., 1970). Each participant was presented with one of three sets of eight messages, and immediately after watching each message, participants had to classify it as either a lie or truth. Finally, participants were thanked, paid, and debriefed.

### Results

#### Truth Bias

**FIGURE 3:**
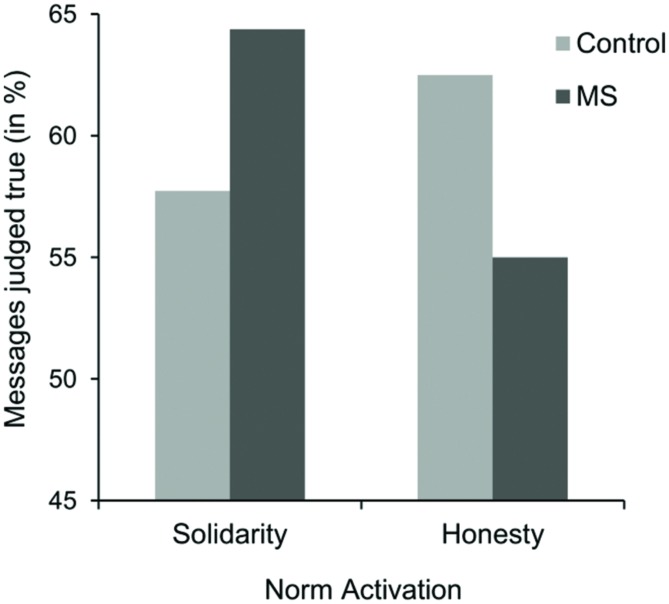
**Means of messages judged true (in %) as a function of norm activation and salience condition in Study 3**.

Overall, participants classified 59.9% (*SD* = 14.62) of the messages as true. This value is significantly different from 50%, *t*(80) = 6.08, *p* < 0.001. Results of the 2 (Salience: MS vs. dental pain control condition) × 2 (Norm activation: honesty vs. solidarity condition) ANOVA on number of messages judged true (in %) revealed no main effects, both *F*s < 1. However, as predicted, a significant interaction effect of salience and activation condition occurred, *F*(1,77) = 4.87, *p* = 0.030, $\eta_{p}^{2}$ = 0.06. As expected, when the importance of honesty was pronounced, percentages of messages judged true were not significantly different from 50% in the MS condition (*M* = 55.00%, *SD* = 14.85), *t*(19) = 1.51, *p* = 0.148, whereas the truth bias occurred in the dental pain control condition (*M* = 62.50%, *SD* = 14.62), *t*(19) = 3.82, *p* = 0.001 (see **Figure 3**). Although these results are in line with our hypotheses, simple effects analyses revealed that percentages of messages judged true in the honesty condition were not significantly lower in the MS condition compared to the dental pain control condition, *F*(1,77) = 2.71, *p* = 0.104. As in Study 2, we would like to note that the *p*-value is based on a two-sided test. Thus, given our directional hypothesis, the difference appears to be at least marginally significant. Furthermore, percentages of messages judged true in the solidarity activation condition were significantly different from 50% in the MS condition (*M* = 64.38%, *SD* = 15.85), *t*(19) = 4.06, *p* < 0.001, as well as in the dental pain control condition (*M* = 57.74%, *SD* = 12.17), *t*(20) = 2.91, *p* = 0.009. Again, simple effects analyses revealed that percentages judged true in the solidarity activation condition were not significantly higher in the MS condition compared to the dental pain control condition, *F*(1,77) = 2.17, *p* = 0.144. Additionally, simple effects analyses revealed that percentages of messages judged true in the MS condition were lower in the honesty condition compared to the solidarity activation condition, *F*(1,77) = 4.23, *p* = 0.043, *d* = 0.61. No significant effect of norm activation occurred within the dental pain control condition, *p* = 0.294.

#### Detection Accuracy

**FIGURE 4:**
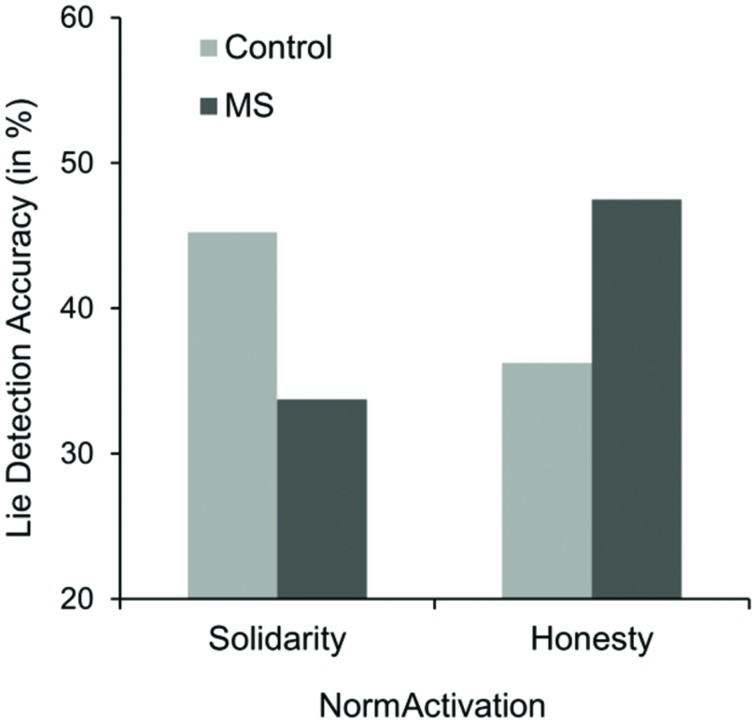
**Means of detection accuracy of actual lies (in %) as a function of norm activation and salience condition in Study 3**.

Overall, the mean percentage of correct lie-truth classifications was 50.62% (*SD* = 16.76). On average, participants were not better than chance in their lie-truth classifications, *t* < 1. Results of the mixed-model design 2 (Salience: MS vs. TV control condition) × 2 (Norm activation: honesty vs. no-activation control condition) × 2 (Type of message: lie vs. truth) ANOVA on detection accuracy (in %) revealed a main effect of the type of message, indicating that participants were better at classifying actual truthful messages as true (*M* = 60.49%, *SD* = 20.87) than they were at classifying actual deceptive messages as lies (*M* = 40.74%, *SD* = 23.53), *F*(1,77) = 38.25, *p* < 0.001, $\eta_{p}^{2}$ = 0.33 (veracity effect). Most importantly, however, the ANOVA yielded the predicted three-way interaction, *F*(1,77) = 4.87, *p* = 0.030, $\eta_{p}^{2}$ = 0.06. Simple effects analyses for detection accuracy for actual lies within the honesty condition revealed that participants under MS were marginally significantly better (*M* = 47.50%, *SD* = 26.78) compared to the dental pain control condition (*M* = 36.25%, *SD* = 23.61), *F*(1,77) = 3.05, *p* = 0.085 (see **Figure 4**). Additionally, and in line with our argument that activating different norms should be responsible for this effect, simple effects within the MS condition showed that participants in the honesty condition were also significantly better at detecting actual lies (*M* = 47.50%) than were participants in the solidarity condition (*M* = 33.75%), *F*(1,77) = 4.55, *p* = 0.036. Simple effects within the solidarity condition indicated that participants under MS were marginally significantly better worse (*M* = 33.75%, *SD* = 18.63) than in the dental pain control condition (*M* = 45.24%, *SD* = 23.21), *F*(1,77) = 3.26, *p* = 0.075. Norm activation within the dental pain control condition revealed no significant effect, *p* = 0.166. Regarding detection accuracy for actual true messages, no effects occurred (accuracy ranged from 57.50 to 62.50%), all *F*s < 1.

#### Mediation of Detection Accuracy

The regression analyses supported the hypothesized mediation of percentage of messages judged true on detection accuracy of actual lies. The interaction of Salience (dental pain control condition = -1, MS condition = 1) × Norm activation (solidarity = -1, honesty = 1) predicted the percentage of messages judged true in step one, *b* = -3.53, SE *b* = 1.60, *t*(77) = -2.21, *p* = 0.030. In step two, the percentage of messages judged true predicted participants’ detection accuracy, *b* = -16.23, SE *b* = 1.96, *t*(76) = -8.27, *p* < 0.001. In step three, the total effect of the Salience × Norm activation interaction on detection accuracy of actual lies, *b* = 5.69, SE *b* = 2.58, *t*(77) = 2.20, *p* = 0.031, was reduced to non-significance when accuracy was regressed on the interaction and the percentage of messages judged true, *b* = 1.76, SE *b* = 1.95, *t*(76) = 0.91, *p* = 0.368. Additionally, bootstrapping the proposed mediated moderation (using Model 8 of the Process macro of Hayes, 2013, and 5000 samples) also revealed significant results, that is, the 95% confidence interval did not include zero [0.99, 15.92].

### Discussion

Results of this study show additional support for our hypothesis that by activating the norm of honesty, MS decreases the truth bias when judging potentially deceptive messages of others. By contrast, when solidarity was pronounced, MS increased the truth bias. Mediational analyses further indicated that these effects affect the accuracy rate when judging actual lies: when people less (more) frequently judged messages as true rather than as false, they automatically improved (worsened) in correctly judging actual deceptive statements. Unexpectedly, as in Study 2, no effects on actual true messages occurred. Additionally, and again parallel to Study 2, comparing the judgmental bias within the TV control condition reveals that norm-activation affects truth bias by trend. Compared to the honesty activation condition, we would have expected that in the solidarity condition, the truth bias is at least as equally strongly developed (without MS). Although this trend was not expected, it should not be overstated because the effect is not significant.

## General Discussion

The current studies addressed the societally important issue of existential threat on veracity judgments. By referring to research on TMT (e.g., McGregor et al., 1998; Jonas et al., 2008), which has indicated that MS increases the sensitivity toward the compliance of cultural norms, especially when they are in focal attention, in both studies, we predicted and found evidence for the idea that when the importance of honesty is explicitly pronounced, MS leads to a reduced truth bias what (mathematically contingent) led to better detection accuracy regarding actual lies. Additionally, in Study 3, explicitly activating the importance of group solidarity increased the truth bias after MS what again led to worse detection accuracy regarding actual lies. This last prediction was based on the well-supported idea that MS increases ingroup bias (e.g., Greenberg et al., 1990).

By applying signal detection theory (Green and Swets, 1966), we assumed motivational changes in judgmental error weighing to be responsible for effects on judgmental biases (Hurst and Oswald, 2012). The truth bias is based on the normally high aversion toward the error of judging an actual message as a lie (error 1). Reducing the weight of this type of error should therefore lead to a reduced truth bias. In Study 1, we found evidence for this assumption, supporting the supposed underlying process of error weighing. Given that MS has been evidenced to increase people’s need for salient cultural norms to be fulfilled, activating the honesty norm in a situation where dishonesty is present should increase a defensive reaction after MS (e.g., Rosenblatt et al., 1989). Based on this, when the norm of honesty was activated, participants in the MS condition perceived error 1 as less serious compared to the control condition. Although we did not asses perceptions of error 2, we would predict that error 2 is perceived as more serious after honesty activation and MS. By contrast, when it is highly important to bolster group solidarity, avoiding error 1 becomes highly important. The best strategy then is to refrain from accusing people of lying. In line with this notion, in Study 3, solidarity activation under MS increased the truth bias.

Although the findings fit to our theoretical considerations, we’d like to discuss an alternative hypothesis, which is how MS might influence bias in judgments of veracity: one might assume that honesty activation and MS increase the truth bias instead of decreasing it, as we suggested and found. Given that MS enhances the need of an anxiety-buffering shelter, one could assume that when activating the norm of honesty, MS strengthens the belief that the world is a trustworthy place, where deception does not happen. Hence, most statements should be judged as true. Evidence on MS induced reduction of dissonance (Jonas et al., 2003) and increased self-serving bias (Mikulincer and Florian, 2002; Rudert et al., 2015) could be interpreted as support for this kind of positive illusion. Although seemingly plausible, we claim that such a perspective, in general, has its evolutionary limits and is therefore less valid. Rudert et al. (2015), for example, found that people reduce their regrets after being reminded of their own death. However, as the authors stated themselves, motivational and also (negative) affective states evolutionarily developed to enable an adequate adaptive reaction (Smith and Semin, 2004). That means, as feelings of regret can be assumed to serve an evolutionary function (e.g., by inducing the motivation to learn from mistakes), ignoring this state in the long run can be considered to be rather detrimental. Thus, although MS might contribute to beliefs in positive illusions in the short term, it is doubtful (and also not evolutionarily functional) that human beings are unlimitedly able to construct the world according to their motivation of feeling safe and happy (cf. Berger and Luckmann, 1967). If this is the case, then MS would less likely increase the motivation to actually engage in worldview validation. For example, activating pro-environmental norms would not lead to increased pro-environmental behavior (as evidenced by Fritsche et al., 2010), but rather would lead to the perception that the environment is not suffering at all. Beyond this contradicting line of argument, we further want to mention that we did not use a subtle priming (cf. Jonas et al., 2008), but in contrast, explicitly emphasized the norm’s societal importance. Additionally, we suggest that the role of experimental scenario plays a crucial role in our findings. Introducing participants to judge potentially deceptive messages implies a violation of the norm of honesty and increases suspicion. Thus, we activated honesty by highlighting its societal importance in a situation where dishonesty was present. We therefore predicted a defensive reaction of increasing skepticism (i.e., reduced truth bias). However, it is not clear to what extent the current findings depend on this specific situation. Hence, the current findings should only be cautiously transferred to everyday situation where no initial reason for suspicion is given.

Looking at actual true messages, we predicted honesty activation and MS to reduce accuracy mathematically due to an increased truth bias (e.g., Levine et al., 2010). However—despite the existent effects in truth bias—in both studies, accuracy on actual true messages remained unaffected by MS and norm activation, indicating that variations in truth bias did not affect variations in detection accuracy of lies and truths to the same degree. We speculate that this can be ascribed to the fact that the percentage of messages judged true were only moderately reduced and did not fall below the 50% level (i.e., there was no lie bias; see also McCornack and Levine, 1990). Furthermore, it should be mentioned that the truth bias found in experimental settings (where people are forced to make a judgment) is likely to be underestimated compared to interactions outside the lab (Bond and DePaulo, 2006).

Whereas Studies 2 and 3 provided support for our interaction hypothesis, in both studies, the honesty norm activation did not reduce truth bias without MS. At first glance, this seems to be in contrast to traditional approaches (e.g., Cialdini et al., 1991). However, our data aligns with many studies which failed to find effects of simple norm activation, but which did find an effect on people’s attitudes and behaviors only when additional motivation (e.g., MS) came into play (e.g., Gailliot et al., 2008; Jonas et al., 2008; Fritsche et al., 2010; Schindler et al., 2013). Taking a closer looking at the interaction patterns further revealed that honesty activation withouth MS by trend even results in an increased truth bias. Although those differences are not significant, they are contrary to our expectations. In Study 2, the trend is probably due to the missing truth bias in the baseline condition (i.e., no-activation in the TV control condition). In Study 3, compared to the honesty activation condition, we would have expected that in the solidarity condition, the truth bias is at least as equally strongly developed (without MS). Given those rather puzzling, unexpected trends, the validity of our conclusions should be considered with caution. Nevertheless, we think that the two studies provide convincing evidence for our idea that the truth bias is affected by existential threat, depending on honesty norm activation.

Although our findings indeed show existential threat to affect truth bias and detection accuracy of actual lies, we did not find any support for effects on overall detection skills, as this would imply higher accuracy rates for detecting lies and truths as well. Research provided strong evidence that improved detection skills especially relate to the use of verbal information, which requires a certain degree of motivation and capacity (Reinhard, 2010; Reinhard et al., 2011b, 2013; Reinhard and Schwarz, 2012). Based on this, there should be certain circumstances in which MS might also lead to increased detection skills. Given that TMT research evidenced MS to increase self-esteem striving (e.g., Pyszczynksi et al., 2004), one could assume that if accurately detecting deception is linked to a boost in self-esteem (e.g., a judge who wants to fulfill the ideal of finding the truth), MS should increase the motivation to accurately distinguish between false and true statements, leading to higher detection accuracy. Parallel to this reasoning, Schindler and Reinhard (2015) recently found MS to increase deception accuracy for people with a dispositional, strong belief in a just world. The authors assumed such people to be highly concerned about matters of justice (such as accurately judging the veracity of a message). Therefore, people with a strong belief in a just world were assumed to be highly motivated to accurately detect deception when there is reason to engage in just behavior (in this case, MS). Given that MS appears to affect overall detection accuracy as well as judgmental biases, future research should systematically investigate the boundary conditions for this mixed pattern of results.

Returning to the political incident mentioned at the beginning of the introduction, our findings appear especially interesting in cases of existential threats, such as the events of 9/11, as it seems likely that political leaders pronounce norms of solidarity. According to our results, this might result in less suspicion toward the sender’s messages, whereas pronouncing the importance of being honest instead might lead to a higher probability of increasing skepticism.

## Conflict of Interest Statement

The authors declare that the research was conducted in the absence of any commercial or financial relationships that could be construed as a potential conflict of interest.

## Footnotes

1 In this study, as well as in Study 2 and 3, data was checked for MS effects on affect and, consistent with prior research on TMT, no significant effects were found, all *ps* > 0.056.

2 Additionally, we used signal detection analysis for hypothesis testing (e.g., Stanislaw and Todorov, 1999). Signal detection analysis generally yields two parameter estimates: response bias *c* (criterion; i.e., a measure of true/false response tendencies) and discrimination ability *d′* (d prime; i.e., a measure of the ability to detect truths/lies). Parallel to analyses of percentage of messages judged true, analyses for Study 2, *F*(1,152) = 4.12, *p* = 0.044, as well for Study 3, *F*(1,77) = 4.65, *p* = 0.034, revealed significant interaction effects on *c*. In both studies, no significant effects occurred on *d′..*

3 Note that the same *F*-value occurred when analyzing the three-way interaction on detection accuracy. This is due to the perfect correlation between percentage of messages judged true and the differential score of lie detection and truth detection accuracy, leading to the assumption that in our case detection accuracy does not depend on any detection ability (see Study 3).
